# Research Progress on the Biological Activity of Ganoderic Acids in *Ganoderma lucidum* over the Last Five Years

**DOI:** 10.3390/life14101339

**Published:** 2024-10-21

**Authors:** Siyi Wang, Longyu Wang, Jiaolei Shangguan, Ailiang Jiang, Ang Ren

**Affiliations:** Sanya Institute of Nanjing Agricultural University, Key Laboratory of Agricultural Environmental Microbiology Ministry of Agriculture, Department of Microbiology, College of Life Sciences, Nanjing Agricultural University, Nanjing 210095, China; 2023116051@stu.njau.edu.cn (S.W.); nausanya@njau.edu.cn (L.W.); 2019216016@njau.edu.cn (J.S.)

**Keywords:** ganoderic acids, bioactivity, pharmacological mechanism

## Abstract

*Ganoderma lucidum* (*G. lucidum*) is a traditional edible and medicinal mushroom in China. The main bioactive components in *G. lucidum* include triterpenoids, polysaccharides, steroids, and sterols. Ganoderic acids (GAs) are one of the most abundant triterpenoids found in *G. lucidum*, garnering significant attention from researchers in the fields of medicine and health care. We summarize the extensive studies on the physiological function of GAs in anti-cancer, anti-inflammatory, radiation protection, anti-aging, liver protection, anti-microbial, and neuroprotection areas, among others. This review provides a comprehensive overview of the recent advances in the bioactivities and pharmacological mechanisms of GAs, aiming to delineate the current research directions and the state of the art in this field. This analysis helps to rapidly identify new bioactivities of GAs and understand their mechanisms, leading to more effective treatments for various diseases.

## 1. Introduction

*Ganoderma lucidum* (*G. lucidum*) is a white rot fungus belonging to the *Basidiomycota* phylum, the *Agaricomycetes* class, the *Polyporales* order, and the *Ganodermataceae* family [1]. *G. lucidum* has been used as medicine in China for thousands of years. The earliest record is found in the *Shennong’s Classic of Materia Medica*, the first pharmacological work of the Eastern Han Dynasty in China [2]. *G. lucidum* has many beneficial effects on human health and is known in folklore as the elixir of life and immortal grass. The *Compendium of Materia Medica* records that *G. lucidum* tastes bitter, is non-toxic, and can nourish the body and enhance wisdom [3].

*G. lucidum* has considerable nutritional and health properties, and is revered as a valuable medicinal mushroom. The extraction of various bioactive constituents from the fruiting body, spores, and mycelium has extensive therapeutic functions, revealing the profound potential of *G. lucidum* in the fields of medicine and pharmacology. For example, the polysaccharides of *G. lucidum* modulate the immune system and ameliorate inflammatory responses, showing promise in the treatment of chronic inflammatory diseases [4,5]. The triterpenoids from various medicinal fungi such as *G. lucidum*, *Poria cocos* and *Antrodia camphorata* have been reported to have a wide range of biological properties, such as anti-cancer activity against breast, prostate, and lung cancer cells [6,7]. These triterpenoids, consisting of six isoprene units, are mainly categorized into two classes: pentacyclic and tetracyclic triterpenes. They can be further differentiated into C30, C27, and C24 groups based on the number of carbon atoms in their molecules, with a relative molecular mass ranging from 400 to 600 [8]. The identification of these constituents has significantly broadened the horizon for the application of medicinal fungi in anti-tumor therapies. In addition to polysaccharides and triterpenoids, *G. lucidum* also contains a spectrum of other bioactive substances, including steroids, sterols, and so on [9,10,11]. This intricate chemical composition underpins the broad pharmacological activities of the mushroom and provides a substantial basis for its diverse therapeutic applications.

**Figure 1:**
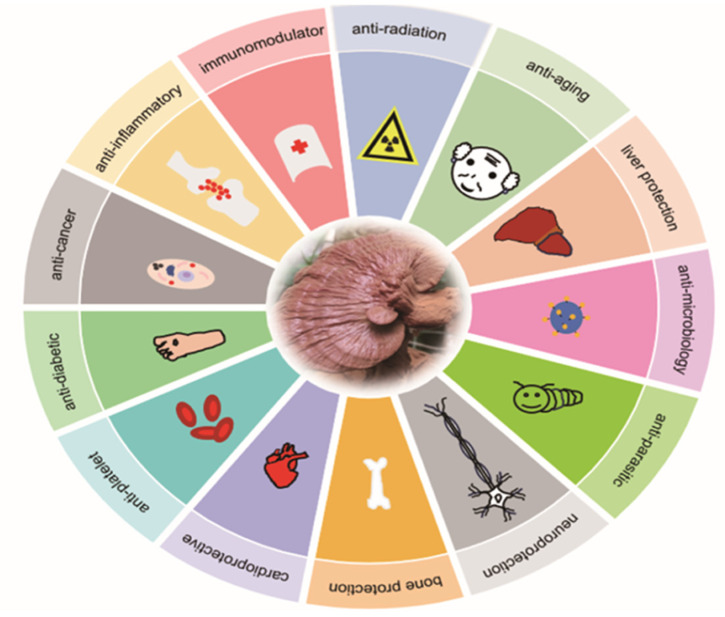
Diverse pharmacological actions exhibited by GAs.

Ganoderic acids (GAs), as the main component of triterpenes in *G. lucidum*, are highly oxidized lanostane-type triterpenes, including ganoderic acid A (GA-A), ganoderic acid B (GA-B), ganoderic acid C (GA-C), ganoderic acid DM (GA-DM), ganoderic acid D (GA-D), ganoderic acid T (GA-T), and others [12,13]. Statistical evidence indicates that the main functions of GAs include anti-cancer, anti-inflammatory, immunomodulator, anti-radiation, anti-aging, liver protection, anti-microbiology, anti-parasitic, neuroprotection, bone protection, cardioprotective, anti-platelet, and anti-diabetes activities [4,13,14,15,16,17,18,19,20,21,22] (Figure 1).

**Figure 2:**
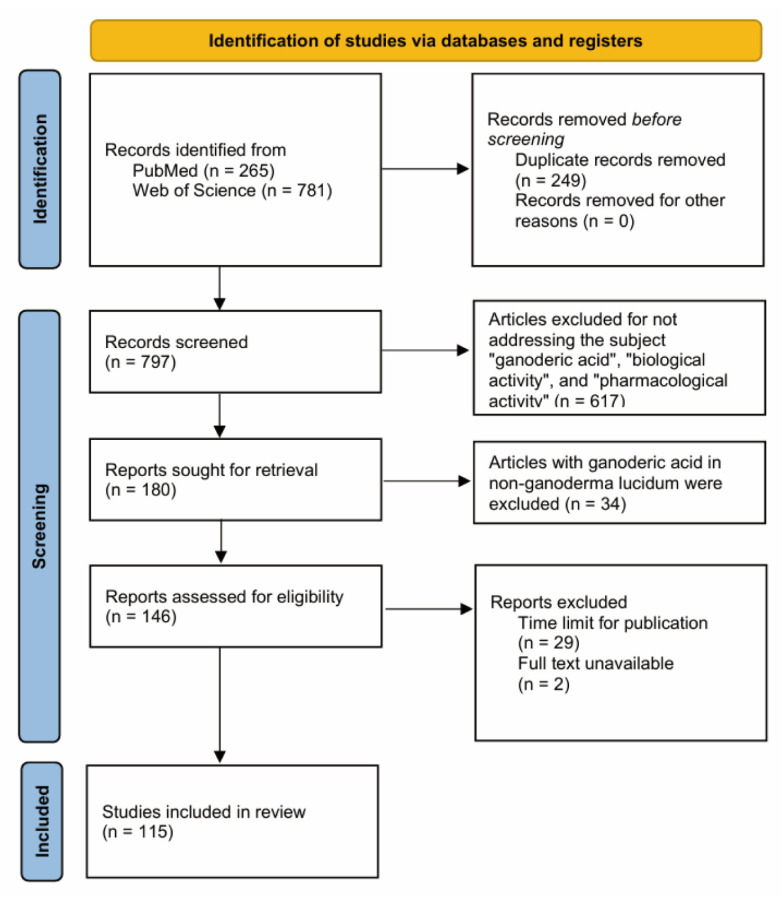
Flowchart of the article selection process for GA-related articles.

This review provides an overview of the research progress on GAs by summarizing experimental papers published over the past 5 years (from 2020 to 2024) using the PubMed and Web of Science databases with the keywords “ganoderic acids”, “biological activity”, and “pharmacological activity”. Additionally, it includes review articles published over the past decade (from 2014 to 2024) using the same databases with the keyword “ganoderic acids” (Figure 2). The primary objective is to provide a comprehensive synthesis of the existing literature on the biological activities and pharmacological mechanisms of these secondary metabolites, thereby providing a holistic and up-to-date perspective on the research directions in this specialized field. This work will accelerate the discovery of novel bioactive properties of GAs, revealing new horizons in the therapeutic potential of these compounds and laying the groundwork for the development of more effective and targeted therapeutic strategies against a wide range of diseases.

## 2. Anti-Cancer

Cancer represents a significant social, public health, and economic challenge of the 21st century, accounting for the majority of mortality cases attributed to non-communicable diseases (NCDs) globally [23,24,25]. A substantial body of experimental research has been conducted on the application of GAs across a range of cancer types, including lung cancer [26,27,28], breast cancer [29,30], prostate cancer [31,32,33], liver cancer [34,35,36,37,38,39], cervical cancer [40,41,42], neuroblastoma [43], and human glioblastoma [44]. In recent years, the evolution of scientific technology has facilitated novel discoveries regarding the anti-cancer properties of GAs, thereby enhancing our comprehension of the underlying mechanisms of action in oncology.

The research team has demonstrated that GA-DM can effectively trigger and enhance autophagic and apoptotic processes in A549 and NCI-H460 non-small cell lung cancer (NSCLC) cells. This is achieved by exerting inhibitory effects on the PI3K/Akt/mTOR signaling cascade, thereby exerting a suppressive effect on cancer progression [45]. GA-A and GA-DM have been demonstrated to suppress the growth of anaplastic meningioma by modulating N-myc downstream-regulated gene 2 (NDRG2) and altering intracellular signaling pathways, thereby reducing tumor volume, inhibiting in vitro cell proliferation, and enhancing in vivo survival rates. These findings suggest that they may have therapeutic potential for the treatment of meningiomas [46]. GA-D downregulates the expression of phosphorylated proteins in the mTOR signaling pathway, including PI3K, AKT, and mTOR, thereby synergistically promoting apoptosis and autophagic cell death in esophageal squamous cell carcinoma cells, achieving effective adjuvant anti-cancer activity [47].

Moreover, research has focused on enhancing the anti-cancer efficacy of GAs through combination with other materials. For instance, the conjugation of the anti-human epidermal receptor 2 (HER2) monoclonal antibody (A. Her2) with the nanocarrier PMBN (poly [MPC-co-(BMA)-co (MEONP)]), followed by the loading of GA-A, has been demonstrated to enhance the inhibitory effect on the proliferation of HER2-overexpressing breast cancer cells [48]. Polymeric nanoparticles (PNP) loaded with tamoxifen (TF) and GA-A exhibit superior anti-cancer activity in the 7,12-dimethylbenz(a)anthracene (DMBA)-induced rat mammary tumor model [49]. The combination of GA-A with a protease-activated targeting chimera has been demonstrated to exhibit optimal anti-tumor activity against p53-mutant breast cancer in both in vitro and in vivo models. This is achieved by the degradation of the murine double minute 2 (MDM2) protein and the subsequent upregulation of p53 and p21 [50].

## 3. Anti-Inflammatory

**Figure 3:**
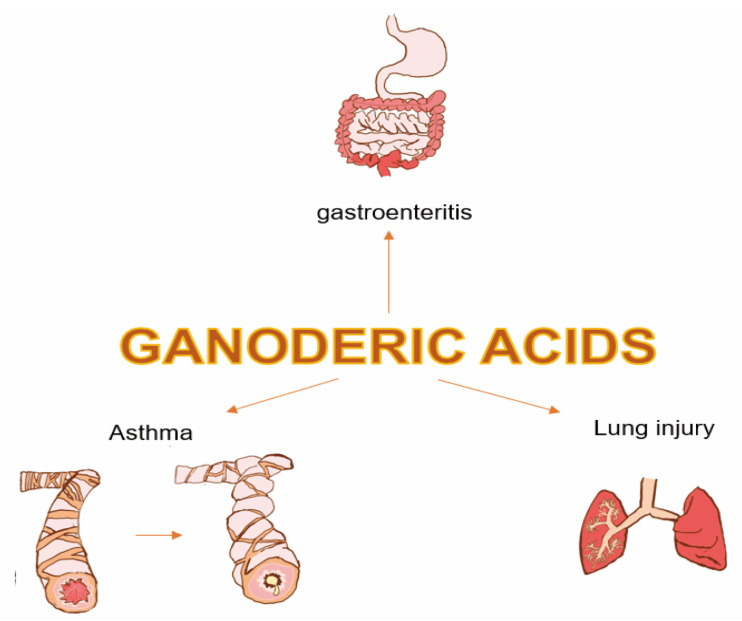
Diverse actions exhibited by GAs in anti-inflammation.

Inflammation is one of the most potent weapons of the immune system, which is initiated to eliminate inflammatory agents when the body encounters invasion by harmful organisms. It is an essential regulatory factor for maintaining homeostasis within the body. However, excessive or persistent inflammatory responses can promote the development of various chronic diseases [51]. There is substantial literature reporting the anti-inflammatory effects of Ganoderma polysaccharides, while studies on the anti-inflammatory effects of GAs are relatively scarce, such as in chronic gastrointestinal inflammatory diseases [52], asthma [53], and lung injury protection [54,55] (Figure 3). In recent years, exploration of the therapeutic potential of GAs for chronic inflammatory diseases has gradually increased [56].

### 3.1. Anti-Atherosclerosis

**Figure 4:**
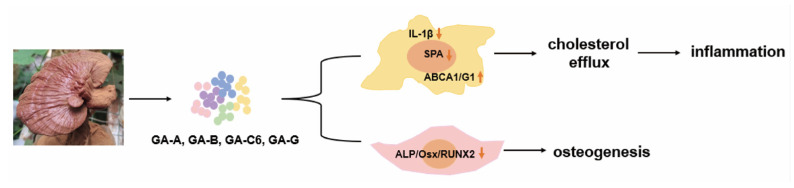
GA attenuates atherosclerosis by specific mechanisms. The upward arrow (↑) indicates that the addition of ganoderic acid leads to an increase in the expression of ABCA1/G1, while the downward arrow (↓) indicates that the addition of ganoderic acid results in a decrease in the expression of IL-1β, SPA, and ALP/Osx/RUNX2.

Atherosclerosis (AS) is a chronic inflammatory disease characterized by the infiltration of lipids and the formation of plaques in the walls of blood vessels. Through the application of mouse models and in vitro experimentation with macrophages, it has been revealed that GAs can preserve the stability of atherosclerotic plaques and elicit anti-atherosclerotic effects. This is achieved through the modulation of the TLR4/MyD88/NF-κB signaling pathway, which results in the inhibition of M1 macrophage polarization [57,58]. Subsequent studies further reveal that GA-A can inhibit inflammation and lipid deposition inhuman monocyte (THP-1) cells induced by oxidized low-density lipoprotein (ox-LDL) through the Notch1/PPARγ/CD36 signaling pathway, providing theoretical guidance for the therapeutic application of GA-A in AS [59]. Furthermore, in Zheng et al.’s study, GA-A, B, C6, and G are found to promote cholesterol efflux mediated by ABCA1/G1, thereby inhibiting inflammation in macrophages, and to suppress the osteogenic function mediated by RUNX2 in vascular smooth muscle cells (VSMCs), thus alleviating atherosclerosis [60] (Figure 4).

### 3.2. Anti-Arthritis

GA-A has demonstrated a significant therapeutic effect on the inflammation of the tarsal joints in collagen-induced arthritis (CIA) rat models, through the modulation of the JAK3/STAT3 and NF-κB signaling pathways [61]. Similarly, it has been demonstrated to improve osteoarthritis by inhibiting the secretion of matrix metallopeptidase 13 (MMP-13) through the relative expression of the nuclear factor kappa-B ligand (RANKL)/osteoprotegerin (OPG) ratio [62]. Subsequent studies have further revealed that it can slow the progression of osteoarthritis by alleviating endoplasmic reticulum stress and blocking the NF-κB pathway [63].

### 3.3. Anti-Asthma

Asthma is a heterogeneous airway inflammatory disease associated with inflammation driven by T helper 2 cell (Th2) cytokines and non-Th2, TNF-α-mediated inflammation. GA-A exerts an anti-asthmatic effect by inhibiting the TLR/NF-kB signaling pathway, reducing inflammatory cells, decreasing the expression of interleukin-4 (IL-4), IL-5, IL-13, and TLR/NF-kB signaling proteins, thereby alleviating the inflammatory response in bronchial asthmatic mice [64]. GA-B suppresses the production of IL-5 by inhibiting GATA3 while maintaining the expression of Foxp-3 and T-bet genes, enhancing the levels of IL-10 and Interferon gamma (IFN-γ), thus reducing the incidence of allergic asthma [65]. GA-C1 achieves the goal of treating asthma by reducing pulmonary inflammation and neutrophils, inhibiting the levels of TNF-α, IL-4, and IL-5, decreasing MUC5AC gene expression, and reducing the production of reactive oxygen species (ROS) [66].

### 3.4. Anti-Neuroinflammation

Neuroinflammation plays a pivotal role in the initiation and progression of neurodegenerative diseases. Microglial-mediated neuroinflammation has been identified as a primary cause of neurodegenerative disorders. GA-A has been demonstrated to exert a pronounced inhibitory effect on lipopolysaccharide (LPS)-induced neuroinflammation in BV2 microglia by activating the farnesoid X receptor (FXR), inhibiting the release of pro-inflammatory factors, and promoting the expression of the neurotrophic factor BDNF [67]. Similarly, deacetylated GA-F, a strategic structural modification, enhances its bioactivity by potentiating its anti-inflammatory capabilities. This alteration enables deacetylated GA-F to effectively target neuroinflammation in microglial cells through the NF-κB pathway, thereby providing a therapeutic approach to treating neuroinflammation [68].

Multiple sclerosis (MS) is an inflammatory demyelinating disease of the central nervous system, representing a major cause of non-traumatic neurological dysfunction in young individuals. GA-A modulates neuroimmunity by enhancing anti-inflammatory and regenerative markers such as IL-4 and BDNF, while suppressing inflammatory markers such as IL-1β and IL-6, and activating an FXR-dependent mechanism to ameliorate neuroimmunological imbalance and promote remyelination in MS [69].

### 3.5. Anti-Intervertebral Disc Degeneration

The primary cause of low back pain is intervertebral disc (IVD) degeneration, with nucleus pulposus (NP) cells being the most significantly affected. GA-A can alleviate the dysfunction of NP cells by modulating the NF-κB pathway to reduce inflammation and extracellular matrix (ECM) degradation induced by IL-1β, thereby treating intervertebral disc degeneration [70]. In the study conducted by Wang et al., GA-A mitigates IVD degeneration by inhibiting the TLR4/NLRP3 signaling axis to suppress apoptosis, oxidative stress, and inflammatory responses in NP cells induced by H_2_O_2_ [71].

## 4. Anti-Radiation

The impact of GA-T on radiation sensitivity is investigated utilizing HeLa cells that have been exposed to gamma radiation. It has been demonstrated that GA-T not only induces apoptosis in ionizing radiation-exposed HeLa cells but also induces necrosis as the concentration of GA-T increases, which may be related to the inhibition of caspase 8, depletion of ATP, and increased production of ROS [72]. Using a model of lens epithelial cells (LECs) exposed to UVB radiation, it has been discovered that GA-A offers protective effects on SRA01/04 cells and rat lenses by enhancing cell viability and antioxidant activity, activating the PI3K/AKT pathway, inhibiting apoptosis, and delaying cataract formation [73].

## 5. Anti-Aging

In recent years, GAs have achieved significant success in the cosmeceutical field, with triterpenoids from *G. lucidum* frequently appearing in cosmetic formulations [51,74]. They are employed as photoprotective agents to regulate excessive pigmentation and suppress inflammatory skin diseases. However, the mechanisms of action and biological targets underlying their beneficial effects on dermatology are not yet fully understood. It has been demonstrated that GA-A induces the proliferation of keratinocytes, accompanied by an increase in the expression of cyclin-dependent kinase proteins, and a significant increase in migration rate and activation of tissue remodeling factors has been observed. Additionally, it exhibits antioxidant effects, providing evidence for the anti-aging properties of GAs and its potential development as a cosmeceutical skin product [75].

**Figure 5:**
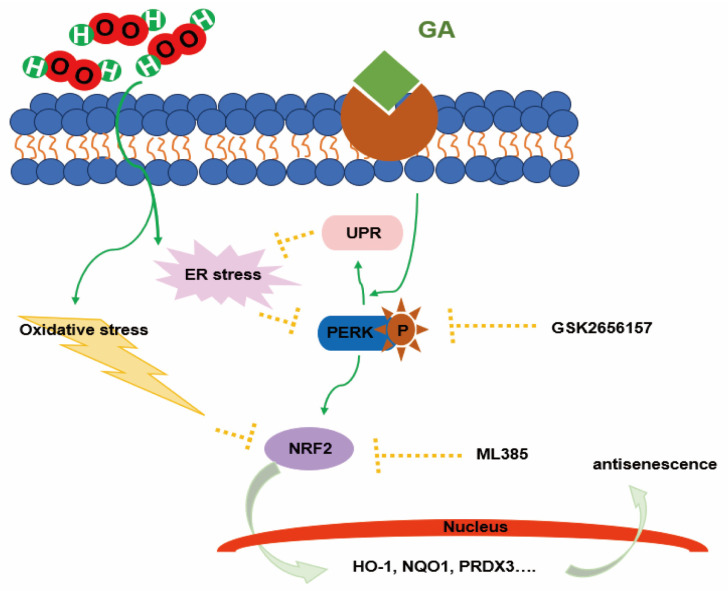
The proposed antisenescence mechanism of GA-D in hAMSCs.

Aging is a significant risk factor for the development of many chronic diseases. The senescence and exhaustion of adult stem cells are considered hallmarks of organismal aging. Using a model of oxidatively stressed, senescent human amniotic mesenchymal stem cells (hAMSCs) induced by hydrogen peroxide in vitro reveals that GA-D delays hAMSC senescence by activating the endoplasmic reticulum kinase (PERK)/nuclear factor-erythroid 2-related factor (NRF2) signaling pathway [76]. In Yuan et al.’s study, it was further discovered that GA-D targets 14-3-3ε to activate the Ca2+ calmodulin (CaM)/CaM-dependent protein kinase II (CaMKII)/nuclear erythroid 2-related factor 2 (Nrf2) signaling pathway, thereby delaying the senescence of hAMSCs, with 14-3-3ε identified as a target of GA-D [77] (Figure 5).

## 6. Anti-Alzheimer’s Disease

Alzheimer’s disease (AD) is considered to be caused by the accumulation of amyloid-β (Aβ) in the central nervous system for insufficient clearance. Using an Aβ25-35-treated HT22 cell model to establish an AD model, it has been found that GA-A can change the effects of Aβ25-35 in inhibiting cell viability, promoting apoptosis, and senescence, and it has been hypothesized that GA-A delays the senescence of AD cells through the modulation of immune-inflammatory mechanisms via the JAK-PI3K-AKT-mTOR signaling pathway [78]. In a separate study utilizing microglia, it is discovered that GA-A enhances Axl phosphorylation, activates Pak1, boosts autophagy, promotes the clearance of Aβ42, and improves cognitive deficits in an AD mouse model [79]. An imbalance in the Th17/regulatory T (Treg) cell axis exists in the neuroinflammatory process of AD. Using a D-galactose mouse model, it is found that GA-A modulates Treg cells to suppress Th17-induced JAK/STAT signaling pathways, enhances mitochondrial oxidative phosphorylation, and thereby improves mitochondrial dysfunction and alleviates neuroinflammation in AD mice [80].

## 7. Liver Protection

Ganoderic acid XL (GA-XL), GA-AM, and GA-A have hepatoprotective effects [81,82]. In Zhang’s study, it is also found that 12 derivative GAs exhibit significant hepatoprotective activity against H_2_O_2_-induced HepG2 cells [83].

The consumption of mushrooms containing α-amanitin, a representative toxin of the Amanita genus, can cause severe liver injury. GA-A has been demonstrated to prevent α-amanitin-induced liver injury by regulating retinol metabolism, tyrosine and tryptophan biosynthesis, fatty acid biosynthesis, sphingosine biosynthesis, as well as the biosynthesis of spermine and spermidine, and branched-chain amino acid metabolism, exerting a protective intervention [84]. Subsequent studies further discover that GA-A may enhance the survival rate and liver function of mice poisoned with α-amanitin by inhibiting the JAK2-STAT3 pathway, and achieve detoxification [85].

Furthermore, GA-A has been demonstrated to offer protection against both non-alcoholic and against alcoholic liver injury. It improves non-alcoholic fatty liver disease by modulating the levels of free fatty acid synthesis, lipid oxidation, and liver inflammation-related signaling pathways such as NF-κB and AMPK [86]. Furthermore, it ameliorates liver inflammation and fibrosis through oxidative stress and the endoplasmic reticulum stress response [87]. Additionally, it has been demonstrated to improve alcoholic liver injury by enhancing lipid metabolism, modulating the levels of inflammation-associated mRNA, and regulating the gut microbiota composition [88].

## 8. Anti-Microbial

Previous studies have demonstrated that GAs can inhibit the Hepatitis B virus (HBV) [89], Human Immunodeficiency Virus-1 (HIV) [90,91], and Enterovirus 71 (EV71) [92]. In recent years, it has been discovered that GA-T exerts its pharmacological effects against Sendai virus by inhibiting the mTOR signaling pathway, regulating the innate immune system and the inflammatory response to the IL-17 signaling pathway [93].

GA-A has been demonstrated to possess anti-microbial properties. When encapsulated with novel nanoparticles, it exhibits a broad-spectrum anti-microbial effect, demonstrating potent antibacterial activity against drug-resistant Escherichia coli and Staphylococcus aureus [94]. Furthermore, through molecular docking simulations, GA-A, GA-AM1, GA-D, and others have been identified for their activity against Staphylococcus aureus [95]. In addition, GAs have also shown significant anti-microbial activity against Bacillus subtilis [96].

## 9. Neuroprotection

The administration of chemotherapy can result in considerable adverse effects for patients, leading to a notable decline in their overall quality of life. Additionally, chemotherapy can impair various physical and social functions, as well as cause cognitive deficits. GAs can prevent and treat cognitive dysfunction caused by 5-Fluorouracil (5-FU) by improving hippocampal neurons, preventing mitochondrial damage, enhancing the expression of proteins related to mitochondrial biogenesis and mitochondrial dynamics markers in the hippocampus, upregulating the expression of neuron survival and growth-related proteins, and strengthening neuronal function [97]. GAs can also alleviate peripheral muscle fatigue and central fatigue-like behaviors by improving muscle mass and mood damage, inhibiting IL-6 and TNF-α, alleviating the decrease in glycogen content, enhancement of glycolysis, reduction in ATP production, and chronic activation of AMPK, thereby reducing peripheral muscle fatigue. Furthermore, GAs can suppress the TLR4/Myd88/NF-κB pathway and downregulate the expression of IL-6, iNOS, and COX2 in hippocampal tissue [98].

**Figure 6:**
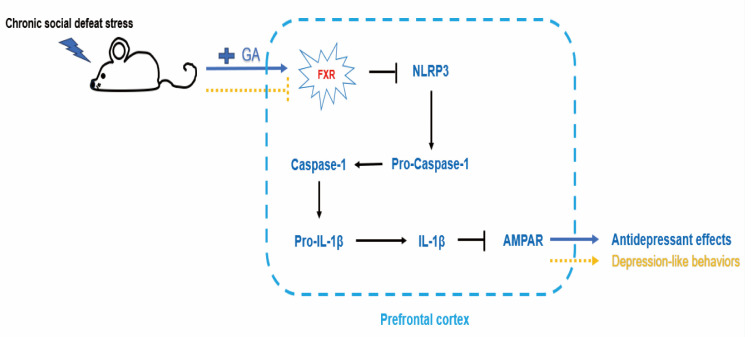
GA-A regulates neuroimmune and antidepressant behaviors.

GA-A has been demonstrated to mitigate the inflammatory response in the hippocampus induced by post-stroke depression (PSD) by activating the ERK/CREB pathway, preventing pro-inflammatory (M1) polarization, and promoting anti-inflammatory (M2) polarization. This has been shown to reduce neuronal damage and depressive-like behaviors in PSD rats, as well as cerebral inflammation [99]. Moreover, it can treat mood disorders and inhibit depressive-like behaviors by modulating the bile acid receptor farnesoid X receptor (FXR), directly inhibiting the activity of the NLRP3 inflammasome, activating the phosphorylation and expression of AMPA receptors, and regulating synaptic activity [100] (Figure 6). Additionally, in Xu et al.’s study, GA-A is found to exert antidepressant properties by affecting the expression of factors related to the regulation of mitochondrial activity and synaptic transmission signaling, providing a new perspective on the antidepressant effects of GA-A [101].

GA-A mitigates the neurotoxin-induced apoptosis of dopaminergic neurons by inhibiting nuclear receptor co-activator 4 (NCOA4)-mediated ferritin autophagy, reducing the neurotoxicity, motor dysfunction, and loss of dopaminergic neurons induced by 1-methyl-4-phenyl-1,2,3,6-tetrahydropyridine (MPTP) and 1-methyl-4-phenylpyridinium (MPP+), thereby alleviating the nervous system and providing a protective effect against Parkinson’s disease (PD) [102]. GA-A can protect neuronal cells (PC) from nitric oxide (NO) stress damage by stimulating β-adrenergic receptors [103]. GA-A significantly inhibits the apoptosis of PC12 cells by downregulating Bax and markedly suppressing the phosphorylation of tau protein at sites S199 and T231, while GA-B also downregulates Bax and Bad, inhibits the activity of glycogen synthase kinase-3β (GSK-3β) to reduce the hyperphosphorylation of tau, and exhibits a better neuroprotective effect on PC12 cells damaged by okadaic acid than GA-A [104].

## 10. Other Functions

GA-A downregulates the Ras/MAPK signaling pathway in a dose-dependent manner, inhibits the expression of proliferating cell nuclear antigen (PCNA), alleviates the growth and progression of renal cysts, and delays the progression of autosomal dominant polycystic kidney disease (ADPKD) [105]. It can also inhibit renal tubular epithelial–mesenchymal transition and hinder renal fibrosis by suppressing the TGF-β/Smad and MAPK signaling pathways, providing a means to block the advancement of kidney diseases [106]. Furthermore, it can improve inflammation, renal fibrosis, and oxidative stress by modulating the Trx/TrxR, JAK2/STAT3, and RhoA/ROCK pathways, thereby protecting the kidneys [107].

In addition to the aforementioned cisplatin, GA-B can reverse ABCB1-mediated multi-drug resistance [108]. GA-D can reverse the resistance to gemcitabine (GEM) in triple-negative breast cancer (TNBC), activating the p53/MDM2 pathway, promoting the ubiquitination and proteasomal degradation of HIF-1α, downregulating HIF-1α-dependent glycolytic genes such as GLUT1, HK2, and PKM2, and significantly reducing the growth of GEM-resistant TNBC cells [109].

In addition, GA-A can also improve hyperlipidemia by regulating liver lipid metabolism, the expression of genes related to bile acid homeostasis, and intestinal flora imbalance [110].

## 11. Limitations

In assessing the review on the bioactivity of GAs, it is important to be aware of some limitations in this area of research. First, although the review may provide some insights into their mechanisms, the specific molecular pathways and signaling networks of many bioactivities are still not fully understood, suggesting that our understanding of how GAs exert their effects needs to be further explored. Second, the review may focus more on research conducted under laboratory conditions, lacking clinical and application data, which limits our understanding of the potential of GAs in practical applications. Finally, the review may not adequately consider the dose–response effects, side effects, and drug interactions of GAs, or fully account for the diversity of population responses, such as the effects of age, gender, and disease status. Therefore, although this review provides valuable resources for understanding the bioactivity of GAs, caution should be exercised in interpreting its conclusions, and the impact of these limitations on research interpretation and future research directions should be recognized.

## 12. Conclusions and Perspectives

Ganoderic acids (GAs) are significant medicinal components reported in the medicinal fungus *Ganoderma lucidum* (*G. lucidum*) [1]. To date, researchers have investigated and reported many biological activities and regulatory mechanisms of *G. lucidum* [4,10,11,12,13,14,15,16,17,18,19]. However, studies on the biological effects and molecular mechanisms of GAs are at a preliminary stage and need further in-depth exploration and investigation.

GAs are a complex mixture from which over 100 different compounds have been isolated to date [4,8]. Among them, GA-A is the most extensively studied and explored. As shown in Table 1, GA-A has potential applications in the treatment of several types of cancer, including liver, prostate, breast, and gastric cancers, as well as protective effects on multiple organs such as the liver, myocardium, and bones. Additionally, GA-A possesses properties like radiation protection, antiviral, and anti-aging capabilities. GAs have a variety of functional groups and side chain structures. The structural diversity of GAs is responsible for their variable pharmacological profiles. For example, in terms of anti-tumor activity, GA-A and GA-B, which are among the earliest triterpenoid compounds isolated from *G. lucidum*, induce apoptosis in tumor cells by compromising the integrity of cellular membrane structures. In contrast, GA-D downregulates the mTOR signaling pathway in esophageal squamous cell carcinoma cells, thereby inducing apoptosis and autophagy. Preliminary studies on the physiological functions of other GAs are also available (Table 1).

The mechanisms by which GAs exert their various biological activities have been confirmed by a large body of research. At the cellular level, GAs exert their effects through precise mechanisms, such as directly targeting cancer cells, interfering with the cell cycle, promoting cellular differentiation and apoptosis, and enhancing oxidative stress responses, thereby combating diseases [13,14,15,16,17,18,19,20,21,22]. The presence of hydroxyl and carboxyl groups in the structure of GAs can modulate their interaction with cell membranes. At the signaling molecular level, GAs modulate the release of inflammatory mediators by interfering with key signaling pathways, including PI3K/AKT/mTOR, NF-κB, Notch1, JAK3/STAT3, and others, thus regulating disease progression. Future research should use advanced methods such as high-throughput screening and molecular biology techniques to further elucidate the therapeutic mechanisms of GAs against various diseases, to investigate their regulatory roles on signaling pathways, and to explore the specificity of these pathways in different diseases.

To date, scientists have developed many precise and effective methods for the clinical application of GAs. For example, the combination of GAs with novel drug delivery vehicles, such as proteolytic targeted chimeras and nanomaterials, has demonstrated the potential to improve resistance to disease treatment. In addition, some studies have optimized the structure of GAs through chemical synthesis, genetic engineering, or biosynthetic strategies to improve the biological activity and targeting selectivity of GAs [48,49,90]. Furthermore, the pharmacokinetic profile of GAs can be optimized through pharmaceutical design and formulation techniques.

There are still some challenges in the clinical application of GAs. Studies have indicated that GA-A inhibits the activities of CYP3A4, CYP2D6, and CYP2E1 in human liver, which may affect the contribution of the hepatic clearance during the metabolism of the drug [111]. Additionally, GAs may cause hypersensitivity reactions in certain populations, exacerbate conditions for patients with asthma and allergic rhinitis, and potentially cause adverse reactions such as rash, paresthesia, abdominal bloating, and dysuria [112,113,114]. In animal models, extracts of *G. lucidum* have shown potential toxic effects on the development of zebrafish embryos [115]. The high lipophilicity of GAs may lead to incomplete dissolution and absorption in the gastrointestinal tract, challenge the distribution to hydrophilic tissues due to biological barriers, and result in a short half-life within the body. These factors contribute to the low bioavailability of GAs [13]. To address these challenges, strategies such as improving their solubility, enhancing permeability, and developing controlled-release formulations can be employed to enhance their therapeutic efficacy. Additionally, it is essential that future studies investigate the synergistic effects of GAs with other drugs or treatment strategies, using multi-drug combination therapies to increase treatment efficacy and reduce side effects. Concurrently, considering the impact of individual differences on drug response, the exploration of personalized medicine strategies will tailor GA treatment based on the genetic background and pathological conditions of patients.

In summary, the prospects for research and application of GAs are promising yet challenging. Through interdisciplinary collaboration and innovative research, GAs have the potential to become an integral component of future medical fields, offering new strategies and methods for the treatment of a variety of diseases.

## Figures and Tables

**Table 1:** The summary table of GAs bioactivities covered in this review.

Compound Name		Biological Activity	Citation Numbers
Ganoderic acid A	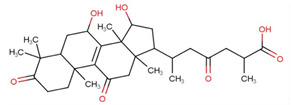	anti-anaplastic meningioma	[46]
		anti-breast cancer	[48,49,50]
		anti-atherosclerosis	[59,60]
		anti-arthritis	[61,62,63]
		anti-asthma	[64]
		anti-neuroinflammation	[67]
		anti-multiple sclerosis	[69]
		anti-intervertebral disc degeneration	[70,71]
		anti-radiation	[73]
		anti-aging	[75]
		anti-Alzheimer’s disease	[78,79,80]
		liver protection	[81,82,84,85,86,87,88]
		anti-microbial properties	[94,95]
		inhibits depression	[100,101]
		anti-Parkinson’s disease	[102,103,104]
		protects the kidneys	[105,106,107]
		improves hyperlipidemia	[110]
Ganoderic acid B	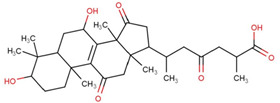	anti-atherosclerosis	[60]
		anti-asthma	[65]
		neuroprotection	[104]
		resistance to drug resistance	[108]
Ganoderic acid C1	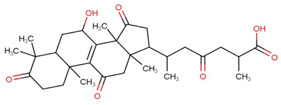	anti-asthma	[66]
		
		
		
Ganoderic acid C6	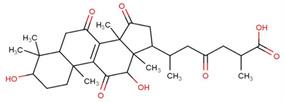	anti-atherosclerosis	[60]
		
		
		
Ganoderic acid D	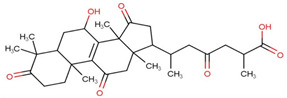	anti-esophageal squamous cell carcinoma	[47]
		anti-aging	[76,77]
		anti-microbial properties	[95]
		resistance to drug resistance	[109]
Ganoderic acid F	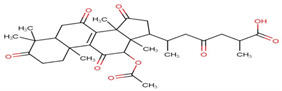	anti-neuroinflammation	[68]
		
		
		
Ganoderic acid G	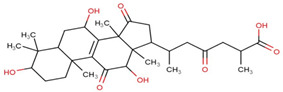	anti-atherosclerosis	[60]
		
		
Ganoderic acid T	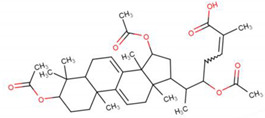	anti-radiation	[72]
		against Sendai virus	[93]
		
		
Ganoderic acid AM	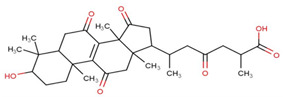	liver protection	[81,82]
		anti-microbial properties	[95]
		
		
Ganoderic acid DM	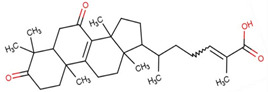	anti-lung cancer	[45]
		anti-anaplastic meningioma	[46]
		
		
Ganoderic acid XL	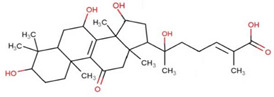	liver protection	[81,82]
		
		
		

## Data Availability

Upon request to the authors M.S.
